# House screening for malaria control: views and experiences of participants in the Roo*Pf*s trial

**DOI:** 10.1186/s12936-022-04321-4

**Published:** 2022-10-21

**Authors:** Caroline Jones, A. Matta, Margaret Pinder, Umberto D’Alessandro, Jakob Knudsen, Steve W. Lindsay

**Affiliations:** 1 KEMRI-Wellcome Trust Programme, Kilifi, Kenya; 2 Centre for Tropical Medicine and Global Health, Nuffield Department of Medicine, University of Oxford, Oxford, UK; 3 Medical Research Council Unit The Gambia at the London, School of Hygiene and Tropical Medicine, Banjul, The Gambia; 4 Department of Biosciences, Durham University, Durham, UK; 5 Department of Disease Control, London School of Hygiene and Tropical Medicine, London, UK; 6 Royal Danish Academy—Architecture, Design, Conservation, Copenhagen, Denmark

**Keywords:** Housing, Malaria, Perceptions, Acceptability

## Abstract

**Background:**

The housing stock of rural sub-Saharan Africa is changing rapidly. With millions of new homes required over the coming decades, there is an opportunity to protect residents by screening homes from malaria mosquitoes. This study, undertaken in the Upper River Region of The Gambia, explores local perceptions of what a good house should provide for its inhabitants and responses to living in a house that has been modified as part of a randomized control trial designed to assess whether improved housing provided additional protection against clinical malaria in children (the Roo*Pf*s trial).

**Methods:**

This descriptive, exploratory study was undertaken over 22 months using mixed-methods (informal conversations, observations, focus group discussions, photovoice, and a questionnaire survey) in a parallel convergent design. Analysis was conducted across the data sets using a framework approach. Following coding, the textual data were charted by a priori and emerging themes. These themes were compared with the quantitative survey results. The nature and range of views about housing and the Roo*Pf*s study modifications and the relationships among them were identified and described.

**Results:**

The data were derived from a total of 35 sets of observations and informal conversations in 10 villages, 12 discussions with the photovoice photographers, 26 focus group discussions (across 13 villages) and 391 completed questionnaires. The study participants described a ‘good house’ as one with a corrugate-metal roof, cement walls (preferably cement block, but mud block covered with cement plaster was also an acceptable and cheaper substitute) and well-fitting doors. These features align with local perceptions of a modern house that provides social status and protection from physical harms. The Roo*Pf*s modifications were largely appreciated, although poor workmanship caused concerns that houses had become insecure. However, the long-term trusting relationship with the implementing institution and the actions taken to rectify problems provided reassurance and enhanced acceptability.

**Conclusion:**

In developing housing to address population needs in Africa, attention should be paid to local perceptions of what is required to make a house secure for its inhabitants, as well as providing a healthy environment.

## Background

Since the turn of the century across rural sub-Saharan Africa there has been a revolution in house construction [1]. Traditional houses constructed using mud walls and thatched roofs are being gradually replaced by houses built of block or mud and cement plaster walls and metal roofs. In most instances, these changes are being undertaken by individual householders rather than by governments or private sector construction companies.

Improvements in housing have, in many areas, coincided with significant reductions of the malaria burden [2] and in areas of endemic malaria, the potential for further reducing transmission through housing improvements such as screening doors and windows has gained increasing attention [3]. Designing appropriate methods and metrics for evaluating the impact of such housing improvements on health and on malaria specifically is currently a matter for debate. Two recently conducted systematic reviews concluded that housing improvements may reduce malaria infection [4–7], resulting in the World Health Organization recommending the use of untreated screening of homes [8].

Alongside evaluating the effectiveness of housing modifications in reducing the malaria risk, several intervention trials have investigated individual and household views on the acceptability of these modifications. For example, a study on the social acceptability of two types of house screening interventions to reduce exposure to malaria vectors was conducted in The Gambia alongside a clinical trial evaluating the interventions’ protective efficacy against malaria [9]. The study found that, besides reporting fewer mosquitoes, participants said that they liked the ceilings and screening as they prevented other insects and small animals from entering their houses and they felt ‘more secure’ in the screened environment. A recently published systematic review and metanalysis of randomized control trials of housing interventions to prevent malaria and *Aedes*-transmitted diseases reported that 11 of their selected studies incorporated a community acceptability component. A key finding was that house screening was perceived to enhance privacy as well as preventing the entry of mosquitoes [10]. These studies provided insights into the acceptability of the housing modifications within a trial context, but little attention has been paid to local perceptions of what features of house construction make a house desirable to live in, aside from the modifications undertaken as part of the trials, or how these ‘disease prevention’ modifications align with how and why house construction choices are being made in any given context.

It is widely recognised that to move from efficacy to effectiveness any disease control intervention needs to be accessible, affordable and acceptable [11, 12]. Should a particular housing modification (or group of modifications) prove to have a demonstrable impact on reducing the burden of malaria, then it is important to understand the extent to which the modifications are not only acceptable in the context of a trial but are aligned with local views on what makes a house desirable to live in and have the potential to be incorporated into local construction practices.

This paper reports on a housing perceptions and experiences study undertaken in a rural area of The Gambia to understand local views about what makes a house desirable to live in; and to explore the experiences of living in a house that had been modified as part of a randomized control trial designed to assess whether improved housing provided additional protection against clinical malaria in children (the Roo*Pf*s trial) [13]. The trial was undertaken in an area of moderate malaria transmission with high coverage of insecticide-treated nets and indoor residual spraying. The RooPfs trial was a two-armed household-clustered randomized control study with 400 households enrolled in each arm across 91 villages with at least two control and two intervention houses per study village [13, 14].

The housing perceptions and experiences study associated with the trial had three key objectives: (1) to explore local perceptions of what a ‘good house’ should provide for its inhabitants and what makes a house ‘bad’; (2) to describe the most valued characteristics of a ‘good house’ and what is required (in terms of construction) to create those characteristics; and (3) to understand if the modifications introduced as part of the Roo*Pfs* trial were considered an improvement and which, if any, had contributed to creating a ‘good house’.

## Methods

### Study setting

The housing perceptions and experiences study was undertaken over 22 months (April 2016 to January 2018) in the Upper River Region (URR) in The Gambia in the context of the Roo*Pf*s clinical trial. The URR is in the far east of the country, where malaria prevalence is the highest [15] and is one of the poorest regions in the country. Malaria transmission is seasonal, from July to December, and peaking in October–November. The rural communities are overwhelmingly farmers, with the predominant ethnic groups in the study villages being Fula (64%) and Mandinka (33%), with 3% ‘other’ [14]. In this area, polygamous marriages are a normative marital institution with a household often composed of multiple houses. Each house within the household is occupied by: (1) a man, (2) wife, usually with their young children, or (3) unmarried male youths. While men are usually the head of the household, the head of a house is often a woman.

**Fig. 1:**
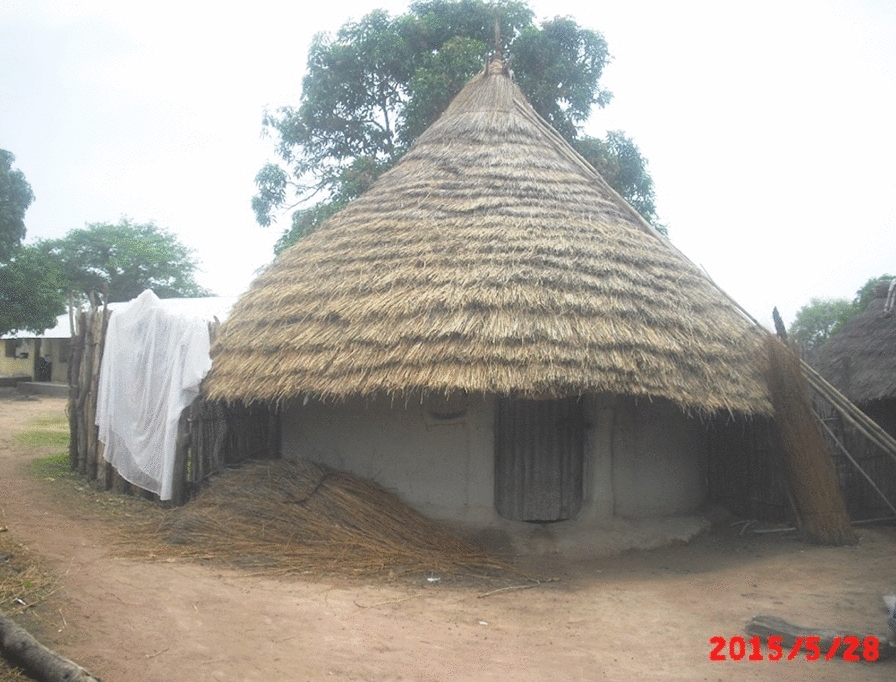
Traditional Round Gambian House

For most of the twentieth century, the predominant rural house construction materials in the URR were mud and wattle or sundried mud bricks for the walls and grass for a thatched roof [16]. Traditional houses were round and constructed with open eaves (the gap between the top of the wall and the over-hanging eaves), two doors (front and back) and sometimes one or two small windows (Fig. 1). Over recent years, in line with the changes that are happening across rural sub-Saharan Africa, thatched roofs in The Gambia are being replaced by corrugated-metal and there is an increasing shift to the construction of square houses using cement blocks for walls [16, 17].

Only ‘traditional’ houses with a thatch roof and open eaves were eligible for recruitment into the Roo*Pfs* trial. There were relatively few of these houses since, in addition to the country-wide shift towards corrugate-metal roofs, the National Malaria Control Programme had, a few years earlier, run a campaign for home owners to close the eaves of houses to reduce malaria mosquito entry. The trial participants had not adhered to this campaign and represented a minority among the general population, often the poorest members communities in a poor region.

**Fig. 2:**
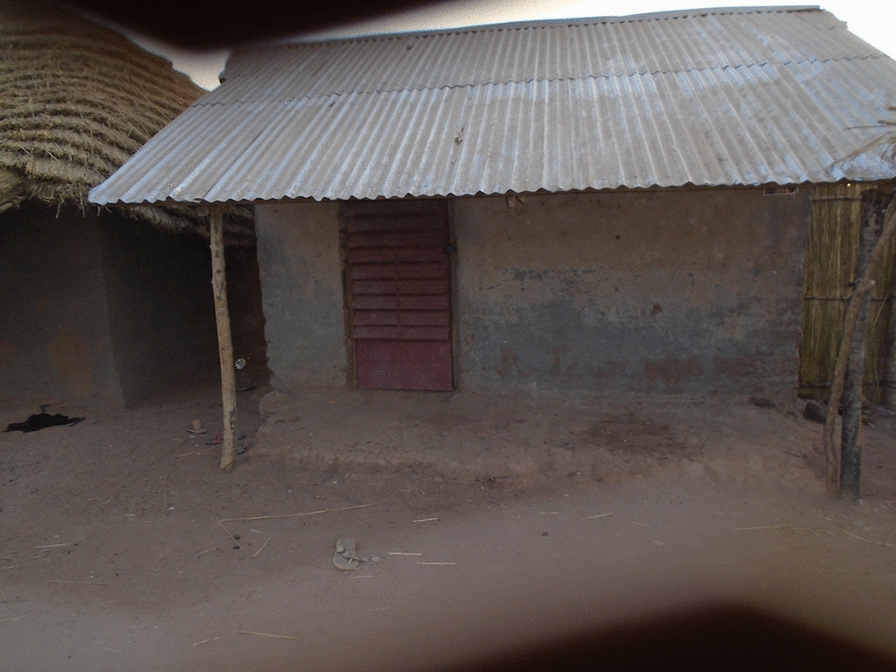
Roo*Pf*s modified house

**Fig. 3:**
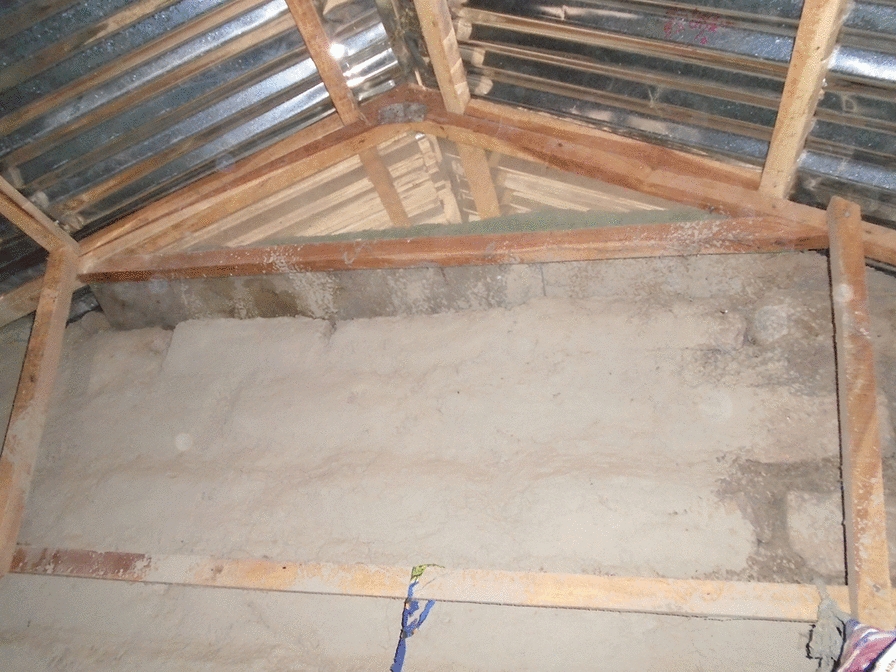
Roo*Pf*s house with gable end window

Prior to the modifications implemented by the trial, all enrolled houses had a single room, thatched roof, open eaves, mud walls in good condition and a front and back door. The Roo*Pf*s housing intervention consisted of removing the thatched roof and installing a corrugate-metal saddle-shaped roof and closed eaves with large-screened windows at the gable ends to help ventilate and cool the house (Figs. 2 and 3).

**Fig. 4:**
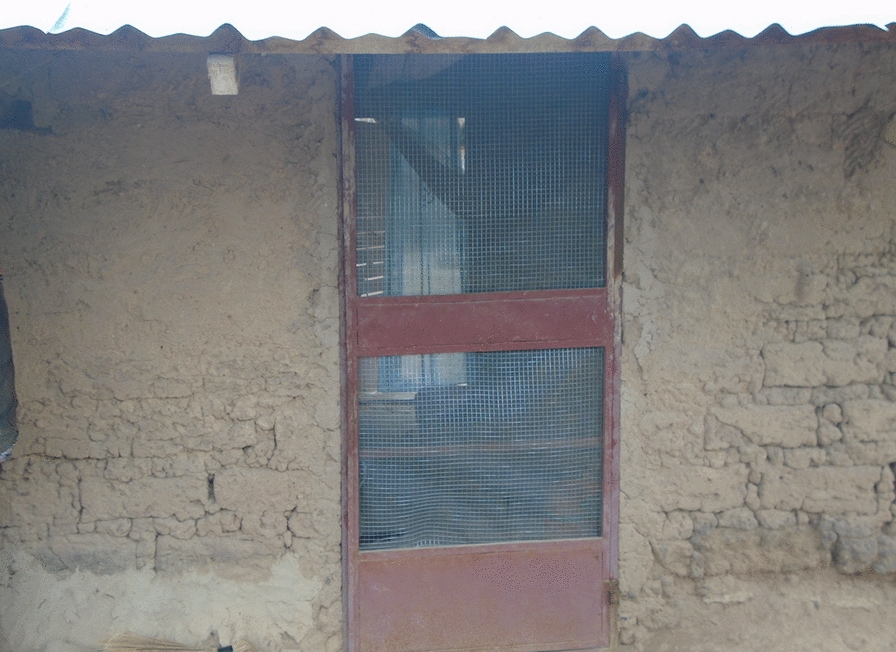
Roo*Pf*s back door

**Fig. 5:**
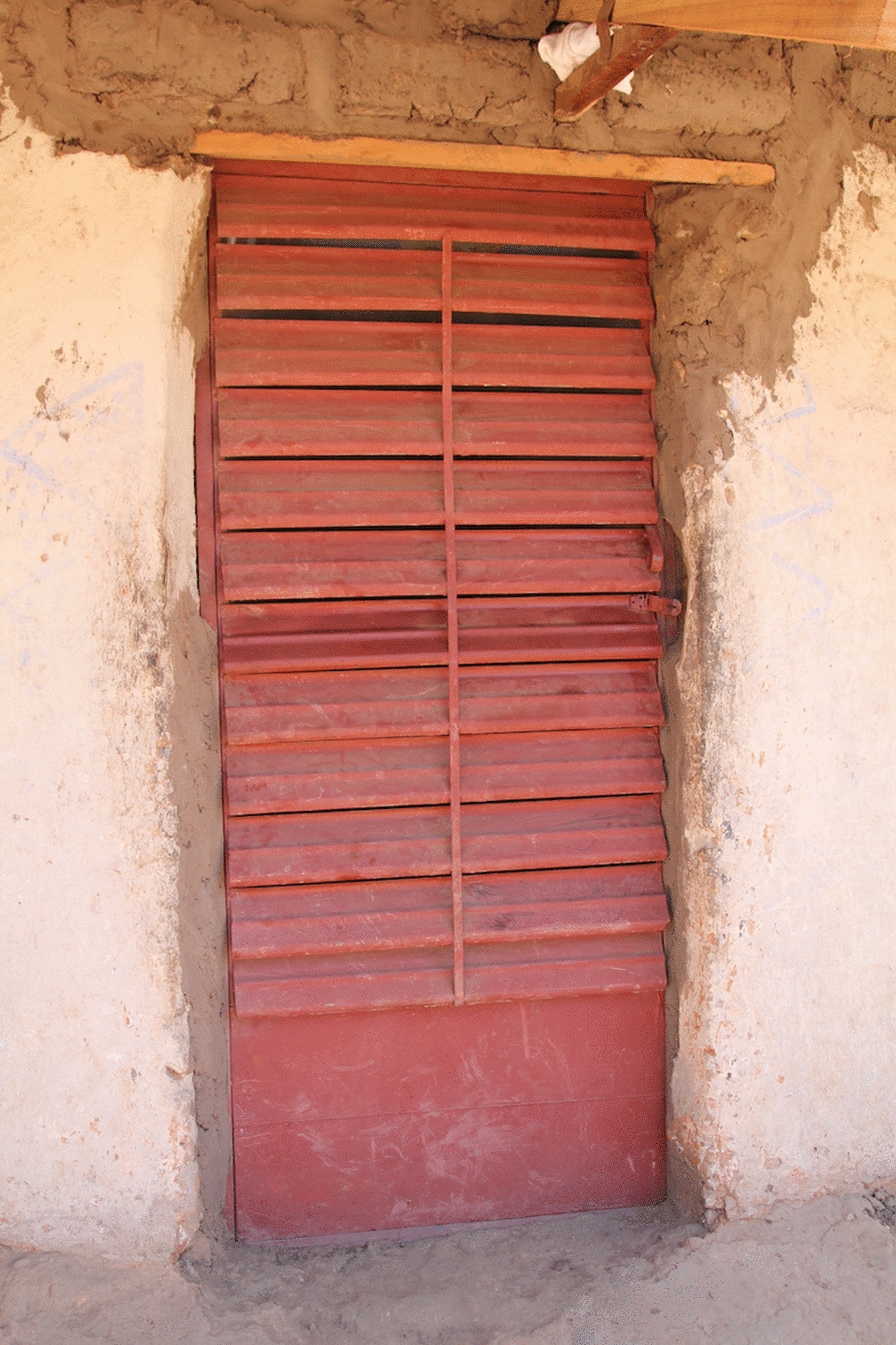
Roo*Pf*s front door

Original doors were also replaced with two screened doors (Figs. 4 and 5).

The intervention used locally available and purchased materials and the housing modifications were undertaken by locally employed masons and carpenters. House modifications were implemented during two dry seasons, from March to June 2015 and between December 2015 to May 2016. In July 2016, the occupants of all enrolled houses (intervention and control) were provided with sufficient insecticide-treated nets (ITNs; Olyset, Sumitomo Chemical, Japan) to cover all sleeping places and there was an ITN distribution carried out by the National Malaria Control Programme (NMCP) in August 2017, as part of the national mass campaign. Indoor residual spraying and seasonal malaria chemoprevention to children 3–59 months old were also provided by the NMCP during the 2016 and 2017 transmission seasons. The Roo*Pfs* clinical study was conducted between June 2016 and December 2017. At the end of the trial, all control houses were offered the opportunity to choose which, if any, of the modifications they would like to have for their house.

### Study design

The perceptions and experiences study reported here was a descriptive, exploratory mixed-method study employing a parallel convergent design [18], using qualitative (informal conversations, observations and focus group discussions—FGDs), participatory (photovoice—PV) and quantitative (questionnaire survey) methods.

### Preliminary studies

Qualitative data collection started with a series of informal observations and conversations undertaken in April 2016 towards the end of the Roo*Pfs* house modification process in the remaining villages where the housing modifications were being completed. These informal conversations were held with the head of the enrolled intervention house, most of them women. While women were frequently the head of the house being modified, they were not necessarily the head of the broader household. This role was usually filled by their husband who, if present, also joined in the conversation. The purpose of this data collection exercise was to explore the roles of house dwellers and household heads in housing decisions, to gauge initial reactions to the modifications being made to the intervention houses and explore local perceptions of what constitutes a ‘good house’. The data from these conversations were used to develop topic guides for subsequent FGDs.

### Focus group discussions

FGDs were held with community members at least three months after the intervention had been completed in their village (Aug/Sept 2016). The FGDs were conducted to: (1) explore perceptions of what a ‘good’ house provides; (2) describe perceptions of the relationship between housing & health; and (3) explore initial perceptions and experiences of living in the modified houses. Participants in the FGDs were purposively selected from among households enrolled in the Roo*Pf*s study. Purposive maximum variation sampling, drawing on data from the clinical trial baseline survey, was used to identify villages and households likely to represent the range of participants involved in the Roo*Pf*s trial (using criteria such as predominant ethnic group in village, geographical location of village and number of households in village enrolled in the trial). Potential participants were approached at their homes by the Medical Research Council the Gambia (MRCG) research unit social scientist (AM) and social science fieldworker and asked if they would be willing/able to take part in a group discussion about their perceptions and experiences of living in a modified house. FGDs were held with one group of men and one group of women per village, between five and eight participants, and in a location and at a time convenient for all participants. Each FGD lasted 60 to 90 min with the discussion conducted in the local language, moderated by AM accompanied by a social science fieldworker. With the permission of the participants, the discussions were audio recorded and additional notes were taken by the social science fieldworker.

### Photovoice

Photovoice (PV) data collection was conducted 12 to 24 months after the house modification (between May and July 2017) among additional villages, selected using the same purposive maximum variation strategy as employed for the FGDs. Photovoice is a participatory visual methodology that allows participants to identify and document objects, process and phenomena of relevance to them in relation to the topic of interest [19]. It has been used widely in public health research over recent years and is increasingly being employed in malaria research [20, 21]. The method involves a series of steps to enhance its ethical use [19]. In April 2017, the potential study villages were visited by the study team and the photovoice activity discussed at a village meeting led by the Alkali (village head). All villages approached in this way agreed to participate in the study and the Alkali, together with members of the village, agreed on the selection of two participants from among the Roo*Pf*s study households (one male and one female) to act as photographers. Following village enrolment, AM and a social science fieldworker returned to each village to carry out a sensitization and training visit lasting two days. During this visit, the two selected photographers received training on how to use the digital cameras and their ethical use. Following the training, the cameras were left in the village with the photographers (usually stored at the house of the Alkali). The photographers were asked to use the following two weeks to take pictures that to them represented a ‘good house’ and a ‘bad house’. They were informed that, when the social scientists returned, they would be asked to upload their photos onto a computer and discuss the pictures taken. During these discussions, the photographers were asked to choose six pictures they agreed best represented a ‘good’ house, a ‘bad’ house and their perceptions of the Roo*Pf*s modified houses. During this visit, following the selection of the six photos, two FGDs were held with participants in the Roo*Pf*s project: one with men and one with women (mixed control & intervention houses). Participants were shown the six photographs and asked to discuss what they thought they represented and whether or not they represent a ‘good’ or a ‘bad’ house and why. The discussions also covered perceptions of how the Roo*Pf*s modified houses compare to ‘good’ houses and the challenges and benefits of living in a Roo*Pf*s modified houses. These FGDs were conducted using the methods described above in the post-intervention FGDs.

### Qualitative data management and analysis

Audio recordings from the FGDs and PV FGDs were transcribed verbatim, translated into English, typed into a Word document and imported into Nvivo 10 for coding and analysis. Transcriptions and translations were undertaken by trained social science research assistants and quality checked by AM. Fieldnotes from the observations and informal conversations were also typed into a Word document and uploaded into Nvivo 10. All identifiers were removed during the transcription process. The selected photographs used in the FGDs were also uploaded into Nvivo 10. Analysis was conducted across the data sets using a framework approach [22]. The coding framework was developed a priori from the study aims and objectives as well as through codes and themes that emerged inductively from the data. The textual data were charted by the emerging themes with the charts subsequently being used, together with the photographs, to map the nature and range of views about housing and the Roo*Pf*s study modifications and identify relationships among them [23].

### Questionnaire survey

Quantitative data collection was undertaken at the completion of the clinical trial in January 2018. The data were collected by fieldworkers from the trial study team as part of a survey conducted among all 800 study houses to assess the condition of the modified houses and to confirm with members of the control houses which modification they would like to receive. Two open questions of relevance to this paper were included at the end of the survey tool for those participants who had been living in the intervention (modified) houses. These were: (1) What do you like about the house modifications? And (2) What do you dislike about the house modifications? The responses were extracted from the main data set and imported into an Excel spread sheet for descriptive quantitative analysis.

The qualitative data were collected before the quantitative data and the two sets of data were analysed independently with the results subsequently compared for triangulation and interpretation [18].

## Results

Qualitative data were collected from 23 study villages with quantitative data collected from all 91 villages recruited for the clinical trial (Table 1). During the data analysis it became apparent that there was little difference in nature and range of issues raised by the two predominant ethnic groups (Mandinka and Fula) and consequently the data in the results are presented across all study villages.

**Table 1:** Data source summary

Method	Number of villages	Number per village	Total
Informal conversations & observations	10 (6F; 4 M)	2–7 (houses modified)	35
FGDs	7 (5F; 2 M)	2 (1 male; 1 female)	14
PV photographers	6 (4F; 2 M)	2 (1 male; 1 female)	12
PV FGDs	6*	2 (1 male; 1 female)	12
Questionnaire surveys	91 [55F; 30 M; 6O]	All trial households	391

FGD is focus group discussion and PV is photovoice

*F* Fula, *M *Mandinka, *O *Other

^*^PV and PV FGDs conducted in the same villages

The questionnaire survey was administered to 391 households. However, for 8 of these households no data were recorded for the two open questions of relevance to this paper. The quantitative results are therefore drawn from 383 responses.

### Perceptions of what makes a house desirable

In the initial informal conversations, FGDs and PV activity, the concepts of a ‘good’ and ‘bad’ house were discussed in terms of the structural qualities of the building and the environment that would exist inside a house built with specific materials and to particular standards. Both the type of construction materials and the quality of the build itself were key in shaping opinions about the likely nature of the internal environment and the desirability of living in any specific type of house.

### Doors

**Fig. 6:**
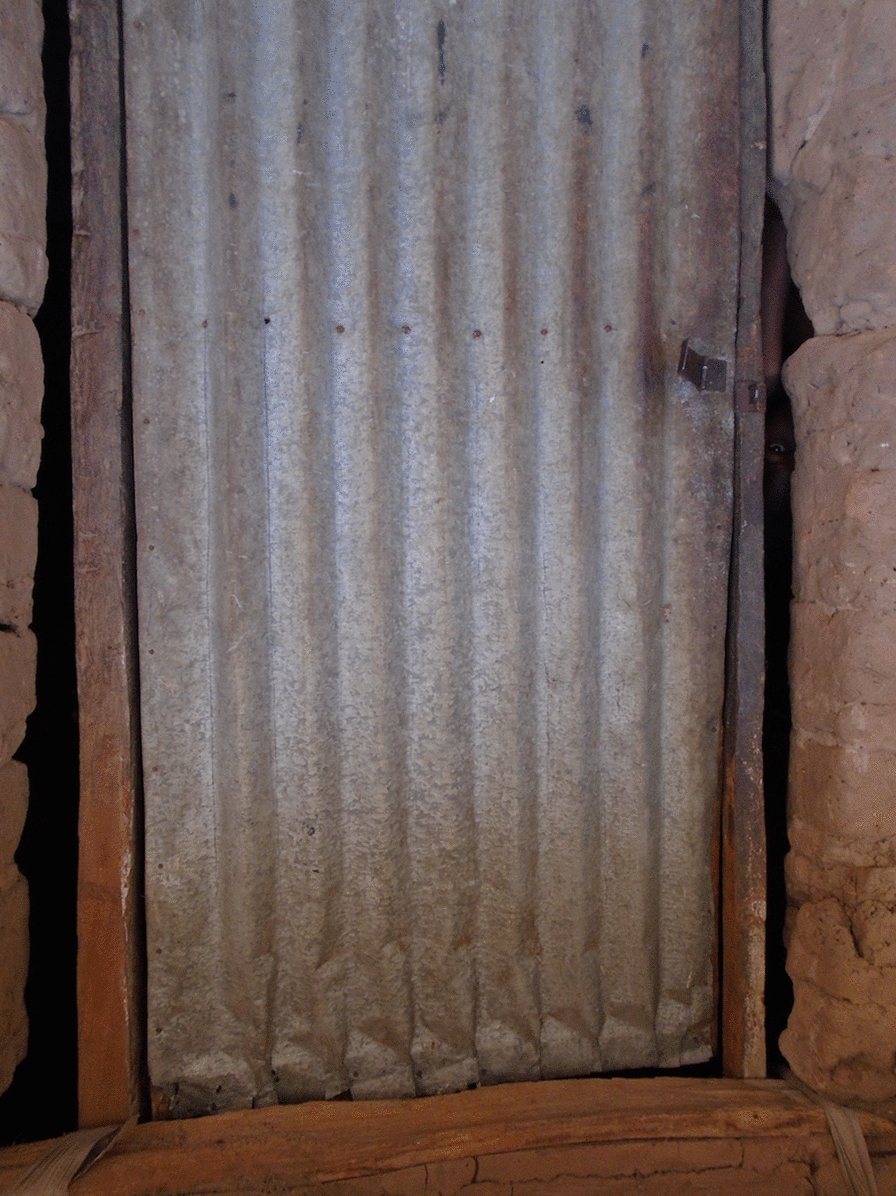
PV Bad door

Doors were a key concern for most participants. In the PV activity, pictures of doors appeared in all sets of photographs taken by the community photographers and were discussed at length in the FGDs and PV FGDs. The door illustrated in Fig. 6 is typical of the pictures taken by the photographers to illustrate a ‘bad’ door.

The key reasons for this categorization were the large gaps between the door and the door frame and between the door frame and the wall. Such gaps were of concern because they would allow the entry of insects and animals:

*When the door is not good you cannot call that house a good one because rats, all types of flies and even a snake can enter the house*. PVFGD9-P4.

The gaps might also allow the entry of people who have not been invited into your house.

*When you have such doors at your house when you lock your door and leave someone can tamper with your belongings in the house during your absence. Someone can come and open the door easily and steal so that makes the house unsafe*. PVFGD3-P1.

Furthermore, many participants across both types of FGD expressed concerns about privacy. If the gaps around the door were too large, then anyone would be able to see inside the house:

*The door had gaps and that shouldn’t be. In a good house you should not be in either side and see the other side through gaps. Your house is your confidential place, but if it is not secured you cannot be protected. You cannot be healthy in such house where by what prevails outside prevails inside. Any house in such a manner is not a good house*. PVFGD2-P9.

As one of the participants said, what is the point of a house without a door?

*when a house has no door is better you sleep in the open. The reason to build a house, roof it and fix doors is for you to be protected but when there are no doors then you are not protected at all.* PVFGD11-P11.

Furthermore, in one of the FGDs a participant mentioned that open doors not only allowed for the entry of human threats but also ‘unnatural’ threats such as demons and spirits;

*Having a good house with a door and a window is safety and prevention. As an old saying amongst Fula elders, if you are inside a house and close the front door and open the back door then “Satan” always enters through the back door. Many evil things come through the door or window but when both are closed then you remain safe inside. FGD3_P21.*

### Walls and roofs

**Fig. 7:**
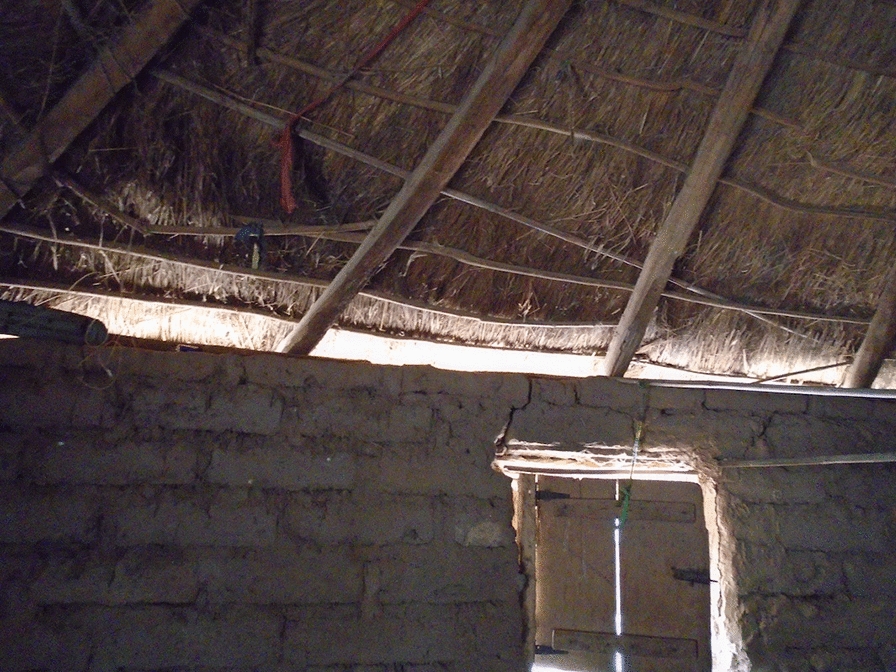
PV Open eaves

The type and state of repair of walls and roofs were also frequently discussed in the FGDs in relation to what makes a good or a bad house. In the PV activity, pictures of roofs and walls (with open or closed eaves; Fig. 7) appeared in all except one of the sets of pictures chosen by the photographers to share and discuss with their community members.

It was generally agreed that cement rendered or plastered walls were a key component of a good house. The primary reasons for this were that such walls would be secure, strong and durable, prevent the entry of insects and small animals, and not allow them to hide in any cracks inside the house.

*When a house is not plastered, not only mosquitoes disturb you but also cockroaches and spiders can all be around to harm you directly or indirectly.* PVFGD2-P10.

*The non-plastered house can accommodate many insects and also the house lack quality. The guarantee of a house depends on cement because if not plastered you cannot be comfortable inside that much.* PVFGD2-P8.

In addition, cement render, or mud plaster made to resemble cement render, was socially desirable and a sign of quality and status:

*When you properly observed the wall* [in the picture] *you’ll think that it is plastered with cement instead of mud. When you don’t have enough money it is more economic if you can plaster it like this…… When you are distant away from the house you’ll think it is plastered with cement whilst it is plastered with mud and sprayed with white sand.* PVFGD3_P1.

Open eaves were universally referred to as a sign of a ‘bad’ house. Key concerns about open eaves were the entry of mosquitoes and other small insects, rats, snakes and dust. An example of a picture taken by the photographers to illustrate their concern with open eaves is shown in Fig. 7, and is discussed in the following quote:

*This house [I] am looking at in the picture is not properly constructed, there’s space between the wall and the roof where mosquitoes and other things can enter through. At the beginning of the raining season there used to be heavy wind blowing and when it finds you in such a house you think that you are outside*. PVFGD2 -P10.

In addition, as with open doors, there were hints in a few of the FGDs that the wind entering through open eaves could bring in ‘other things’ (that may or may not be of natural origin) that could harm the inhabitants:

*The closed eaves are better because when the eaves are left open, dust can enter with lot of things and harm you inside.* FGD8_P52.

In the photovoice activity very few pictures were taken of a thatched roof. Where a picture of a thatched roof was presented, it was shown and discussed as a ‘bad’ house. In all the informal conversations that were held while the house modifications were taking place, as well as in the FGDs and in the PV FGDs, corrugate-metal roofs were seen to be a sign of a good house. Thatched roofs were associated with ‘dirt’, ‘rats’, ‘insects’ and low status.

*It* [a corrugate roof] *can protect us from disease. In a grass house many things can be hiding inside the grass that may harm you, if it’s corrugate nothing can be hidden up there*. FGD6_P42.

In addition, a grass roof required more upkeep and, as one participant explained, it was becoming more and more difficult to find the right kind of grass for the roof:

*A good house is always roofed with corrugate because people prefer corrugate than grass. By having a corrugate roof your husband is free from cutting grass to thatch your house.* FGD4_P27.

Many female participants explained that it was difficult to keep the house clean if you have a thatched roof and they were concerned that a thatched roof conveyed ‘low status’.

…*if my relatives visited me in my thatched house I feel shy due to the bad conditions of the house* …. FGD11_P68.

By contrast having a corrugate-metal roof provided a sense of pride:

*I am very happy to have this house because it is the only corrugate house in the compound…*(Questionnaire respondent).

However, while corrugate-metal roofs were clearly more desirable, a few participants mentioned that thatched roofs tended to be cooler and were heavier so they were less likely to blow away in the high winds during the rainy season. In addition, where grass for the thatch was available it was possible for households to harvest it themselves. Drawbacks associated with a corrugate-metal roof were that it was noisy in the rains, hot in the dry season and expensive.

Table 2 provides a summary of the key benefits and drawback of the different types of roof as described by the participants.

**Table 2:** Benefits and drawback of different roof types

Roof type	Benefits	Drawbacks
Corrugate- Metal Roof	Durable No dust/insects/ Clean Unlikely to leak Higher status	Expensive to buy Hot in dry season Noisy in rains More likely to be blown away
Thatched roof	Cool & quiet Cheap Heavier (doesn’t blow away) Easy to put on a round house	Smells/dirty Not durable (requires frequent changing) Local sticks & grass becoming rare Fire risk

### Clean, tidy, and aesthetically pleasing

The nature of the structural components of the house were discussed, not only in terms of durability and physical security but also in terms of being integral to the ability of its occupants to be able to create a clean, tidy, and aesthetically pleasing environment within the house. Even if the house was structurally sound, an untidy or unclean environment inside the house was discussed as being harmful to health and well-being. Untidy clothes or personal possessions could provide a hiding place for insects (including mosquitoes), snakes and small animals and facilitate the accumulation of dirt and dust, which were seen as harmful to health.

*But when the house is not clean and things scattered all over you won’t notice when scorpion, snake or even mosquitoes enter and hide inside.* PVFGD3_P1.

*When a house is not clean people don’t feel healthy inside. You become inactive when you have visitors and yourself will not be comfortable. A house should be kept clean always*. PVFGD4_P7.

**Fig. 8:**
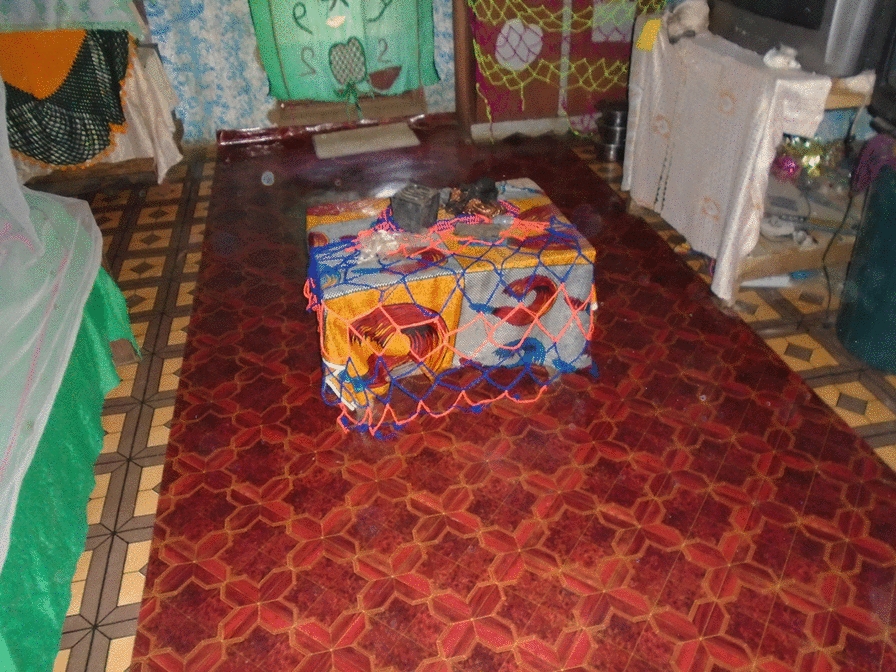
PV inside a good house

In addition, in all three qualitative data sets, the importance of the house being aesthetically pleasing both on the outside and the inside was mentioned as a key feature of a ‘good’ house. Not only was this important for the direct comfort and security of the inhabitants but it was also mentioned as being an important signal of relative wealth. The response to the picture shared in PVFGD2 (Fig. 8) adds support to the idea that the appearance and content of a house are signals of wealth and social status.

*This house is so good that I only aim it for myself. When you have such a house even if you put on ragged cloths people always feel that you have money.* PVFGD2_P7.

In summary, from across the qualitative data sets the key emerging theme was that a good house will provide protection. Protection from natural (wind, rain, dust, light from lightning, insects and animals) and potential supernatural (spirits and devils) hazards as well human-made threats (thieves and criminals); providing a secure environment that is structurally sound, durable, easy to keep clean and aesthetically pleasing. In such an environment people are safe from physical harm and disease and their well-being is enhanced. These attributes were unanimously described as being provided by a well-built structurally sound cement block house with a corrugate-metal roof—a ‘modern’ house in the URR. Such houses are perceived not only to offer more protection from natural and human-made threats, but are also more expensive to construct and as such are an aspirational goal for many of the households involved in the Roo*Pfs* trial. Living in such a house in the Roo*Pf*s trial communities confers social status on the inhabitants, providing a sense of social as well as physical protection.

#### The ‘MRC’ house modifications

The data on perceptions of the modifications to houses carried out in the Roo*Pf*s trial were drawn from the FGDs, the PV activity and PV FGDs and quantitative data from the end of trial survey.

Qualitative data from the informal conversations undertaken while the housing modifications were in progress suggested that while overall the participants were delighted with what they saw being done to their houses, there were a few concerns. The key positive improvements mentioned by the participants were that the houses looked beautiful (aesthetically pleasing) and strong, they had corrugate-metal roofs with closed eaves that would prevent the entry of dirt and insects and the expectation was that they would have more light inside and be easy to keep clean. There were concerns, however, related to the corrugate-metal roof not extending as low down the side of the house as the previous thatch, and the walls being exposed to the elements with potential consequences for the integrity of the mud walls, particularly during heavy rains. Citing the latter, several participants requested cement to render and protect their walls. There were also concerns expressed about the style of the back door (Fig. 4). The key concern was that it was not a solid door but rather a metal door frame with a central bar and covered in netting. It was feared that this door would let in too much light and cold air, and the netting could become torn.

In the subsequent FGDs, PV activities and the questionnaire survey, these positive and negative features of the housing modifications were key recurring themes. In all FGDs held at 3 months after the houses had been modified, participants raised some concerns about aspects of the modifications, while at the same time, expressing their gratitude to the MRCG for investing in improving their houses. While concerns were raised and discussed during the qualitative data collection, the end of study quantitative data suggested that the housing modifications were widely appreciated. Of the 383 participants who had lived in a modified house and provided a response in the questionnaire survey, 56% (214/383) had only positive things to say about the house modifications. An additional 160 participants (42%) had things that they liked and things that they disliked about the housing modifications. For example, many participants said they liked the doors because they reduced the mosquitoes and also the house was cooler at night, but disliked the walls not rendered with cement and so were getting washed away. Just 9 participants mentioned only things they disliked.

The positive responses in the end of trial survey are likely to have been influenced by the maintenance work carried-out during the trial by the local masons and carpenters at the request of the trial team. The trial implementation included a ‘report and repair’ system in which the condition of the intervention houses was assessed during and immediately after completing the modifications (May 2016) and again in August, 2016, and July, 2017 with repairs undertaken in December 2016, June 2017 and December 2017. Residents of houses in the intervention group were also encouraged to report any damage or malfunctioning of the interventions to the nurse field assistants who visited twice per week, from June to December in 2016 and 2017 (malaria transmission season), as part of the clinical trial.

A summary of the likes and dislikes of the modifications as expressed by the participants who had lived in the modified houses and took part in the end of project quantitative survey are provided in Table 3.

**Table 3:** Likes and dislikes of participants living in the modified houses

N = 383	Roof	Doors	Gable windows	Fewer mosquitoes	More Air/ Cooler	Nothing	No data
Liked	55%	30%	0%	31%	3%	3%	2%
Disliked	4%	19%	10%	0%	8%	57%	2%

### Views on the modifications

#### Corrugate-metal roof

In all FGDs the corrugate roof was seen as a positive asset because it meant the householder no longer had to find grass to repair the thatch, the house was lighter and cleaner inside and because of the status that a corrugate-metal roof bestows:

*I am happy with the MRC's support on these housing issues, before in our swampy areas there used to be grass called ‘’Nyantan’’ which we used to roof our houses with and it takes almost ten years before changing it but now due to the low rainfall that type of grass does not grow. Our corrugate roofing does not get moldy and it can serve us for many years before changing it. FGD2_P13.*

*The happiness I have since I occupied the house is so great because before even if my relatives visited me in my thatched house I feel shy due to the bad conditions of the house….Now when my relatives come I don’t feel shy because my house is in good conditions. FGD11_P68.*

**Fig. 9:**
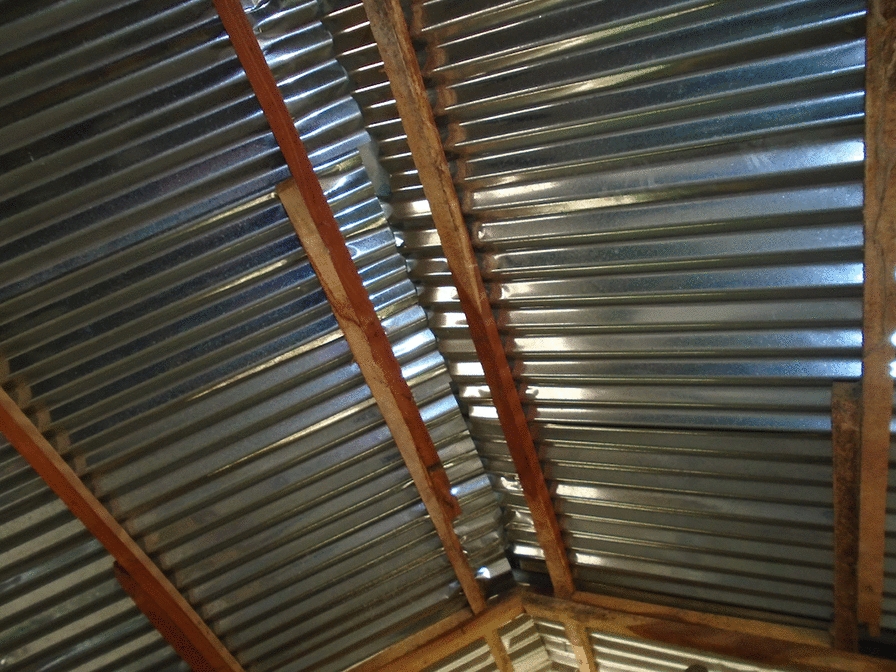
PV Roo*Pf*s corrugate-metal roof

These sentiments were repeated in the PV activities where in all but one of the set of photos chosen by the photographers there were pictures of a corrugate-metal roof. A corrugate-metal roof was universally appreciated and preferred over a thatched roof (Fig. 9).

*In this picture* [Fig. 9] *it is a MRC house and is very good, good roof. Young and old, whoever sleeps in this house can be protected……*. PVFGD9- P2.

The information from the qualitative research was echoed in the responses in the quantitative survey. The most common response to the question: ‘*what did you like about the modified house’* was the corrugate-metal roof (209/383; 55%). Reasons mentioned for liking the corrugate-metal roof were the same as those described in the informal conversations, FGDs and PV activity; that it was more durable and cleaner (less likely to become infested with insects and rats). A few of the questionnaire respondents (n = 15) said that they like the corrugate-metal roof because it was more secure. No one, in either the qualitative or quantitative data collection activities, expressed a preference for thatch over corrugate-metal roofing.

Despite the strong preference for a corrugate-metal roof, many participants in the FGDs and PV activities complained that the corrugate-metal roofs leaked during the rain:

*It is true that in any development there are constrains. My house leaks to the extent that all the beds get wet when raining and we have to move to another house ….* FGD1_P62.

A leaking roof was commonly mentioned as a problem with the corrugate-metal roof during the qualitative data collection activities (which took place in April, August and September 2016 and May and June 2017) but by the end of study survey (January 2018) only 23/383 participants mentioned the leaking as a ‘dislike’. This is likely a consequence of the ‘report and repair’ system that operated during the trial, the last round of which was implemented in December 2017. Participants in the qualitative activities which took place earlier on in the trial are likely to have used the opportunity of having contact with MRCG ‘staff’ to describe aspects of their house that needed attention:

*This is the right time to explain the problems we face with our new houses. We have no right over those carpenters because they are hired by MRC to do the work but we can lodge our complaints to you the MRC staff. FGD2_P20.*

By the end of the trial most leaking roofs had been fixed. Interestingly, of the twenty-three participants who mentioned a leaking roof in the end of trial survey, 15 also expressed a liking for the corrugate-metal roofs and specifically mentioned the corrugate-metal roof in their response to ‘what do you like about the modifications’. For example, a participant said they didn’t like that their roof leaked but also said that “*I like a house of corrugate and thank the MRC for giving me one”.*

While the corrugate-metal roof was clearly appreciated, there were also major concerns about the style of the new roof. A frequent complaint in the FGDs undertaken three months after the completion of the modifications was that the corrugate-metal roofs not only leaked but also did not extend far enough out from the walls of the house to give them protection from the rain.

*MRC did not aim to cause any fault to our houses but hence you asked us to tell you what our constraints are towards the houses then that’s normal. The houses were plastered with mud and the rain has washed it down and presently all the lower parts of the houses are soaked. I do spend the night with my wife and children in the newly roofed house but when raining we move to another house due to the leaking of the roof. Outside is wet, inside is wet and leaking we cannot be comfort in that situation. Presently two of us wanted to abandon our houses and move to another house due to these problems never did MRC aim it that way. We are therefore appealing for your urgent help.* FGD5_P37.

In almost every FGD there were concerns that the mud walls of the houses would be ‘washed away’ by the rain causing their collapse.

*I preferred corrugate roofing*…..[But] *The house should not be odd like the house you modified for us…… The roofing should be lowered and cover some part of the wall to avoid rain water from soaking the wall which may lead to the falling of the wall. If the wall has a problem and fall down that can harm you and your children* …FGD14_P83.

*The only problem with the house is the roof. It’s high on top, rain washes the wall down and as long as that is happening the wall may one day fall on us and that’s why we abandoned our houses.* FGD2_p9.

In two of the FGDs, participants reported that their walls had indeed collapsed:

*The problem with my roof is that it’s high and without veranda. They brought only [one] bag of cement and give it my husband and he told them that the cement won't be enough for plastering. I told them that the reason I am in a thatched house is because of poverty and you see the roof is lowered to protect the wall. They said to me that we will change the house to meet my demands. But when the house is ready the roof is so high without veranda. A bag of cement was given to me to plaster one side of it and as the roof is high rain washed the wall and the house fell down.* FGD14_P83.

To prevent this type of accident happening more frequently, there was a widely held view that the MRC should provide sufficient cement render to apply to all of the mud walls of the houses since it was the modifications that had caused the problems.

*We can roof the houses like the way you did but the reason why we didn’t do it is simply because we can’t afford the cement to plaster the wall. You came and changed the roofs and you did not replace them as they were, conceivably you can have a corrugate house without cement blocks but can’t have one without plastering it with cement. Your carpenters failed to lower the roofs and that caused fear amongst us. Some houses about four to six people sleep in there and that is why in mine when raining I don’t sleep until the rain stops because I always think of the condition of the wall where my family is.* FGD12_P81.

In all of the FGDs, participants requested cement so that they could render their walls and protect them from the rain:

*Only you have power to solve these problems the only thing we can do is to report the matter to you. The reason why you brought corrugate to roof the houses can make you buy cement for the houses too. FGD1_P5.*

In response, the trial team provided one bag of cement to each house that requested assistance with this problem. By the time of the survey at the end of the study, only 26/383 participants mentioned that they still had a problem with the lack of adequate cement render.

#### Doors

During housing modification, several participants were concerned about the doors that were being installed and these concerns were echoed during the FGDs and the PV activity. During these qualitative data collection activities, the front doors were rarely mentioned and few of the photographs taken as part of the PV activity contained images of a front door. Any concerns about the front door related primarily to the way in which it had been installed, with a few participants reporting that there were gaps between the door frames and the walls, or the wooden frames being eaten by termites. By contrast, the back doors were a frequent subject of the photos and a topic of conversation in each of the FGDs.

*Concerning the doors, the front doors are better than back doors and we would like you to change them for us. If you cannot change them, you look for another type of net for back doors as the chicken wire is easily torn even by touching it.* FGD7.

**Fig. 10:**
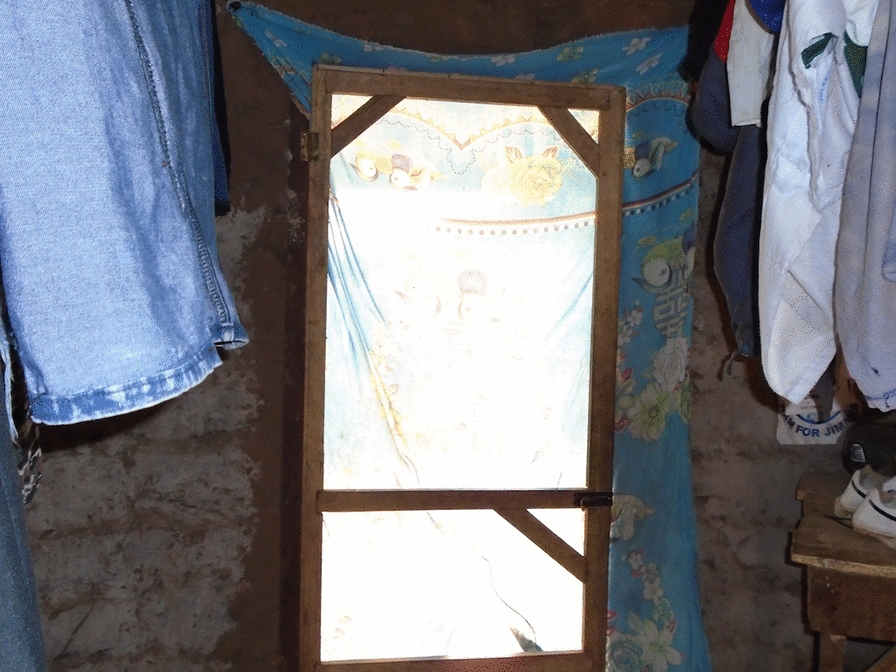
PV Roo*Pf*s backdoor

**Fig. 11:**
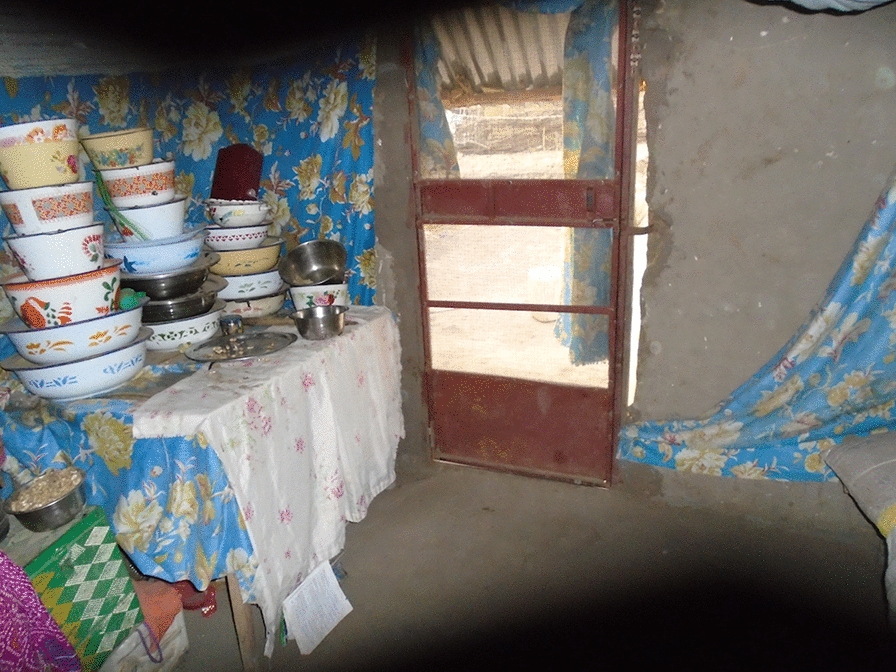
PV poorly installed Roo*Pf*s backdoor

In addition to concerns about the thinness of the netting covering the backdoors that was easily ripped, by animals and children, the main concern about the backdoors was that they were insubstantial and open to dust, light, rain, wind and people. Figures 10 and 11 show pictures of a backdoor taken by one of the PV photographers. In each case participants in the PV FGDs described their concerns about the ‘transparency’ of the backdoors.

*This door* [Fig. 10] *is good and properly fixed but when there is heavy wind dust can enter the house through the door. A good house needs a good protective door which can prevent you from all sorts of harmful things. This door can prevent you from mosquitoes and heat but cannot prevent you from bad people and dust.* PVFGD7_ P5.

*The houses that MRC modified here can prevent you from mosquitoes but when windy you cannot even stand in the house rather to sleep there. The doors can prevent mosquitoes from entering the house but when it is windy and dusty you vacate the house due to the dust.* PVFGD11_ P7.

*The house is good but the door is faulty because it’s a screened door and even when it is raining water enters through*. PVFGD7_P2.

..*but the back door is transparent. The best thing to me when you are inside the house it should be dark and no one outside should see you inside. The way the door is made someone can glance at you inside and see everything clearly. PVFGD7_P7.*

One of the participants in the FGDs said that she had been so worried about the transparent backdoor that her husband had covered it in corrugate:

*Yes, my husband changed it to a corrugate door.* FGD1_P6.

While the style of the backdoor was a concern, the way in which the doors (both front and back) had been installed was also causing some worries. Several of the photographs taken in the PV activity contained doors with large gaps around the frame (Fig. 11), gaps that would allow the entry of mosquitoes and other insects.

*This house in the fifth picture* [Fig. 11] *is somehow good but the only problem is the door which has a gap and cannot close well. …..that gap between the door and the wall if not sealed, not only mosquitoes but also many other things can enter through there to the house*. PVFGD3_P11.

These concerns about the backdoor were, to some extent, echoed in the quantitative data at the end of the study. The most frequently mentioned dislike in the modified houses was the doors (72/383), but at the same time, substantially more participants (114/383) reported that this was a feature of the modified houses that they liked. Among those who reported disliking the doors, there were few concerns about the front door with only 12 participants mentioning concerns that primarily related to security due to ill-fitting doors and problems with termites that were eating the wooden door frames. The back door was more contentious, with 58 of the participants specifically mentioning that they didn’t like the design of the backdoor. This was primarily because they felt that the screen design made the house too cold, it let in too much dust and too much light. There were also concerns about security with a few of the participants mentioning that thieves had entered the house through the backdoor over the previous months.

#### Reduction in mosquitoes and illness and the ITNs

In all FGDs, at least one participant mentioned either that there were fewer mosquitoes in their modified house, or that malaria among their children had reduced or both. This was perceived to be one of the main advantages of the house modifications:

*I am very happy with MRC because they provided us with good houses, free from leakage and the prevalence of mosquitoes reduced. We stay inside and sleep well.* FGD11_P71.

*We are happy with these houses as they have saved us from constant visits to health centres due to malaria treatment.* FGD5_P34.

As part of the trial, all participants were given a mosquito net and it is possible that this might have influenced the perceptions of fewer mosquitoes and less illness. In addition to reporting that they were paying fewer visits to the health facility participants also mentioned the presence of the ITNs:

*We are happy with the houses because all the houses have mosquito nets*. FGD 5_P34

**Fig. 12:**
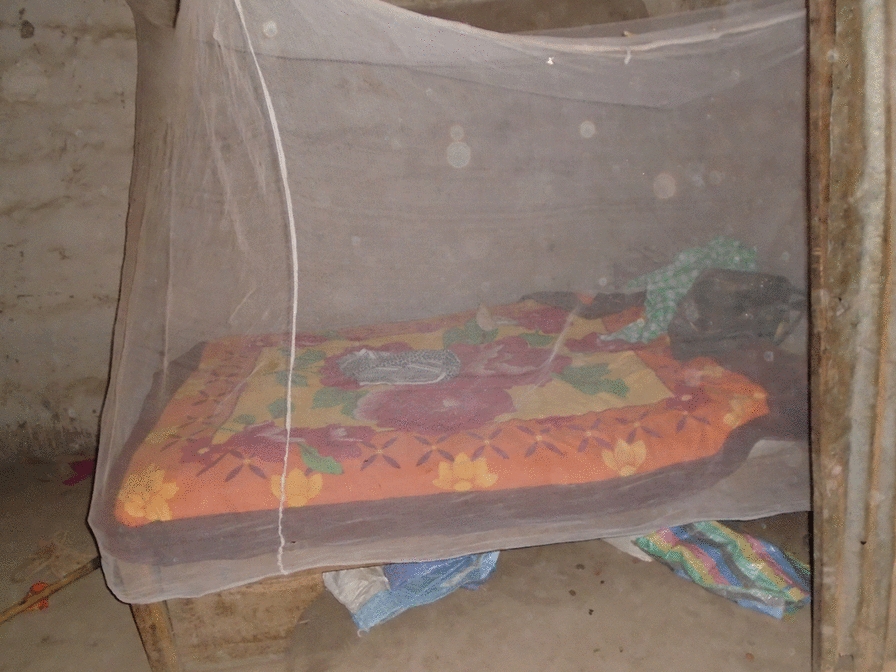
PV ITN over bed

The presence of an ITN in the house was also the most common picture in the PV activity (Fig. 12). The presence of the net was universally perceived to be a sign of a ‘good’ house:

*In this fourth picture the bed is well organised and the net tugged round it. When the bed has net there will be no mosquito interference when sleeping. PVFGD3_P5.*

Similar sentiments were found in the quantitative data. In the survey data the second most frequently mentioned reason for liking the house modification was the perceived reduction in mosquitoes inside the house mentioned by almost a third of the participants (118/383; 31%).

Interestingly, the data from the entomology conducted alongside the clinical trial suggested that the modified houses did not contain fewer mosquitoes than the un-modified, traditional thatch houses. However, enrollment in the trial did provide some participants with access to regular screening for malaria as well as being given an ITN. It maybe that there was less mosquito nuisance while sleeping due to the presence of relatively new ITNs:

*We are happy with the houses because all the houses have mosquito nets……Whenever our children are screened by the nurse they have malaria negative. FGD10_P64.*

In line with the requirements of the ethical conduct of a clinical trial, all participants were taken through the informed consent process, providing them with information on the purpose of the modifications and the trial. This consenting process and additional trial benefits are likely to have had an influence on perceptions of mosquito nuisance and malaria incidence.

*A lot of benefit is gained by sleeping in the houses such as reduction of sickness which is the purpose of the houses*. FGD12_P73.

#### Responsibility for problems

While the overall response to the modifications was very positive, there were some considerable concerns about the type and quality of work that had been undertaken by the masons and carpenters hired by the MRCG to carry out the modifications. That is, problems with the houses were rarely blamed on the MRCG per se, rather they were blamed on the quality of the work undertaken by the contractors.

*MRC knows that though am not saying that the contractors are not qualified but the beneficiaries are not very happy because many doors are not good, termites are grinding the roofing sticks to powder and the roof is leaking*. FGD5_P38.

*I am the one occupying the house but detected some problems there at the roofing level. When they roof the house I told them that water will enter from the corners of the house and they said no. I told them let me go inside and will throw water at the corners and see. We did that and the water entered then I asked them what happened? They say it had entered. When it is raining we know how we slept in our old houses and how we feel the same in the new house too. I told them that some of these houses will fall before rain stops and that’s what happened. I told them that you will finish your work and go leaving us here to suffer with our houses.* FGD12_P78.

Many of the houses required a considerable amount of maintenance, during the trial; fixing leaking roofs, mending the netting on the back doors and repairing poorly installed modifications (doors, gable windows and roofs). However, the report and repair system seemed to help identify and fix most of the problems so that by the end of trial survey most participants were happy with the house modifications and all participants in the control houses requested all modification components for their houses. It was clear from the FGD and PV data that most participants perceived that the responsibility for repairs or amendments lay with the MRCG.

## Discussion

Th Roo*Pf*s housing perceptions and experiences study was undertaken alongside the Roo*Pf*s clinical trial designed to evaluate whether improved housing provided additional protection against clinical malaria among children living in a poor rural region of The Gambia [13, 14]. This study focused on understanding local perceptions of what a ‘good house’ should provide for its inhabitants and what makes a house ‘bad’, and on describing the extent to which the modifications were aligned with local perceptions and which, if any, had contributed to creating a ‘good house’.

In The Gambia, as in many countries in sub-Saharan Africa, the past 70 years has seen changes in the design and materials involved in the construction of rural houses. For example, corrugate-metal was first used as a roofing material in the 1950s and has been gradually replacing grass as the material has become more widely available and householders are able to afford the cost. Mud bricks started to replace mud and wattle (krinting) walls in the 1960s and doors have also evolved with woven materials (bamboo or wattle/krinting) being replaced by one sheet of corrugate metal. The installation of ceilings, facilitated by the changes in roof and wall construction, became more common in rural Gambia in the early 1990s. The closing of eaves was encouraged in a campaign by the National Malaria Control Programme during 2013 following evidence of the impact on malaria [24] Today in the URR, the ‘modern house’ desired by the participants in this study, is one with a corrugate-metal roof, cement walls (preferably cement block, but mud block covered with cement plaster was viewed as an acceptable and cheaper substitute) and well-fitting doors. These features are aspirational for the study participants who were among the poorest households in the one of the poorest regions of The Gambia. Such a house provides visible signs of enhanced social status as well as increased physical security and comfort for the inhabitants. These data suggest that the changes that have been seen over the past 70 years in the Gambia, and elsewhere in Africa, have been influenced not only by social desirability, a modern house being a symbol of economic and social status as the materials need to be purchased rather than collected and constructed by the householder, but also by the enhanced security that houses with corrugate metal roofs and doors and solid block walls offer to their inhabitants. This sense of security is created through protection from the dangers posed to health and well-being by small animals, reptiles, insects and human as well as supernatural threats.

The findings that a ‘well sealed’ house is a desirable house as it offers protection from dust, rats, snakes, and mosquitoes, and helps keep the house ‘clean’ creating a healthy and socially desirable environment, is not new but rather echoes the findings of several other studies undertaken in The Gambia and elsewhere in Africa [9, 10]. In their studies undertaken in The Gambia during the 1980s on the economic and cultural aspects of the use on ITNs, McCormack and colleagues found a clear preference for ITNs made of opaque materials that prevented the entry of rats, snakes and insects, protected against droppings falling from the roof and also provided greater privacy [25, 26]. In these studies MacCormack and colleagues also found that the opaque nets were preferred because they helped protect against owl witches and spirits of the night [25]. A more recent ethnographic study undertaken in the URR and Central River Regions of The Gambia in 2013 and 2014, describes how in Gambian cosmology illness can either be caused by an organism inside the body detectable by biomedicine, or by supernatural forces outside the body that are invisible to biomedicine [27]. These supernatural forces include ‘foul winds’, spirits and witchcraft. A well-sealed house that keeps out the wind is able not only to prevent illnesses caused by the ‘cold’ [27] but also prevent the entry of foul winds and spirits liable to cause illness and other harms. Security and privacy were also found to be important in a recent study undertaken in Tanzania of different types of housing designed to improve health in rural Africa [28]. In this study the timber clad house was preferred over the houses with bamboo or shade net cladding as they were perceived to be more secure, durable and to provide more privacy. In addition, double-storey houses with the sleeping space on the top floor, were preferred as they were perceived to offer greater protection from insects and crawling animals (snakes) as well as being cooler and providing more privacy.

The house modifications undertaken as part of the Roo*Pfs* trial were, in general, perceived to contribute towards creating a good, well-sealed house, with the corrugate-tin roof being most appreciated. However, there were concerns about both some of the modifications and the standards of workmanship. Perhaps unsurprisingly, the key concerns were around the modifications and standards of workmanship that were perceived to make a house less secure. For example, the small overhang of the corrugate-metal roof raised some concerns that the walls were more liable to rain damage. Poorly installed doors created concerns as they allowed the entry of insects, rats and snakes and could not be securely fastened against human or supernatural intrusions. However, by the end of the trial, many of these concerns had been addressed and most householders were happy with their modified house. The participants were all grateful that the MRCG had spent time and money on helping to improve houses, even if the houses weren’t all perfect. It was widely agreed that the MRCG modifications had relieved a financial burden among these poorest with the equivalent re-roofing being unaffordable to trial participants due to price of corrugated metal.

The responses from the participants were overwhelmingly positive about the effects of the modifications on the presence of mosquitoes and malaria even though there was no evidence of this in the clinical trial data [14]. Participant awareness of trial involvement and the nature of involvement can affect participant responses and their perceptions of outcomes [29–31]. The process of trial enrollment creates awareness of the purpose of the trial which is likely to have a significant impact on participant perceptions and behaviours [30]. In this study the participants were aware that the purpose of the trial was to reduce the burden of malaria and so it is perhaps not surprising that several of the respondents in the FGDs reported that the burden of malaria had reduced. In addition, all sets of the PV pictures included a photograph on the inside of a house with an ITN with participants agreeing that a ‘good house’ required an ITN to cover the bed. This is perhaps not surprising when considering all participants were given a mosquito net. Several studies have shown that trust in the implementing organization can have unintended consequences in terms of expectations of the benefits of trial participation [32–35]. In the Roo*Pfs* trial it was clear that the long history of the MRCG in conducting health research in general, and malaria research in particular, had led to levels of trust and expectation among the participants that may have influenced their perceptions of the benefits and effects of the housing modifications. A complex range of factors influence the decision to participate in a trial and these ‘trial effects’ make it challenging to assess the extent to which the reports of trial participants are likely to reflect experience and perceptions under more routine conditions [30]. Nonetheless, at the end of this Roo*Pfs* study, all participants in the control arm requested the housing modifications, suggesting that overall these were acceptable to the study communities.

On current estimates, the population in sub-Saharan Africa will grow by 1.3 billion by 2035 [36]. This growing population will need adequate housing. To help ensure that such housing creates a healthy secure environment and fulfils local criteria for desirability, data is needed on the performance of different housing types as well as information on locally acceptable attributes. The data from this study suggests that in the URR of The Gambia, attributes that create a desirable house for local populations are those that provide a secure environment, but not necessarily a healthy internal one. However, modifications that create a more healthy internal environment but are perceived to decrease the integrity of the structure, or create a less secure environment are likely to be unacceptable to the local population.

## Limitations

A key limitation of the study was that it was conducted in the context of the Roo*Pf*s trial implementation. As discussed, acceptability studies conducted in the context of a trial are likely to be affected both by social desirability bias and by trial effects. These effects were apparent in the data collected for this study (e.g., in the photographs taken in the PV activity which focused on those aspects of a house that were affected by the trial) and were interrogated as part of the analysis and interpretation of the data. While social desirability bias and trial effects were clearly present in the study, the presence of the ‘report and repair’ system encouraged participants to discuss their concerns about their modified houses during the qualitative data collection activities, in the expectation that these concerns would be taken on board and responded to. In addition, triangulation of data from multiple sources (informal conversations, FGDs, PV and questionnaires) and the longitudinal nature of data collection (revisiting communities across a 22-month period) allowed for the identification and interrogation of key recurring themes.

## Conclusions

As the need for new housing in sub-Saharan Africa expands, interventions designed to create healthy houses need to consider local design preferences. In URR, householders were primarily concerned with the security and durability of their homes. Interventions such as replacing a short-lived thatch roof with a more durable corrugate-metal were perceived as providing enhanced security and were universally appreciated. Poorly-fitting doors and screened doors that could easily be torn caused concern. Where house modifications, or new house designs align with what is locally considered to be the features of a desirable house; providing security, built with durable materials and to a high standard, they are likely to be widely welcomed and accepted. However, interventions that make a house feel less secure for the inhabitants are unlikely to be acceptable and householders may make alterations, which potentially decrease the health of the indoor environment but enhance perceptions of security.

## Data Availability

Access to the fully anonymized qualitative and questionnaire survey data requires a formal application to the Scientific Coordinating Committee of the Medical Research Council Unit The Gambia (MRCG) and the Joint Gambian Government's and MRCG Ethics Committee in The Gambia at https://www.mrc.gm/scientific-coordinating-committee/.

## Abbreviations

FGD: Focus group discussion
ITN: Insecticide Treated Net
MRCG: Medical Research Council the Gambia
NMCP: National Malaria Control Programme
PV: Photovoice
URR: Upper River Region
